# Biophysical Characteristics of Successful Oilseed Embryo Cryoprotection and Cryopreservation Using Vacuum Infiltration Vitrification: An Innovation in Plant Cell Preservation

**DOI:** 10.1371/journal.pone.0096169

**Published:** 2014-05-01

**Authors:** Jayanthi Nadarajan, Hugh W. Pritchard

**Affiliations:** Seed Conservation Department, Royal Botanic Gardens Kew, Wellcome Trust Millennium Building, Ardingly, West Sussex, United Kingdom; Lawrence Berkeley National Laboratory, United States of America

## Abstract

Heterogeneity in morphology, physiology and cellular chemistry of plant tissues can compromise successful cryoprotection and cryopreservation. Cryoprotection is a function of exposure time × temperature × permeability for the chosen protectant and diffusion pathway length, as determined by specimen geometry, to provide sufficient dehydration whilst avoiding excessive chemical toxicity. We have developed an innovative method of vacuum infiltration vitrification (VIV) at 381 mm (15 in) Hg (50 kPa) that ensures the rapid (5 min), uniform permeation of Plant Vitrification Solution 2 (PVS2) cryoprotectant into plant embryos and their successful cryopreservation, as judged by regrowth *in vitro*. This method was validated on zygotic embryos/embryonic axes of three species (*Carica papaya*, *Passiflora edulis* and *Laurus nobilis*) up to 1.6 mg dry mass and 5.6 mm in length, with varying physiology (desiccation tolerances) and 80°C variation in lipid thermal profiles, i.e., visco-elasticity properties, as determined by differential scanning calorimetry. Comparisons between the melting features of cryoprotected embryos and embryo regrowth indicated an optimal internal PVS2 concentration of about 60% of full strength. The physiological vigour of surviving embryos was directly related to the proportion of survivors. Compared with conventional vitrification, VIV-cryopreservation offered a ∼ 10-fold reduction in PVS2 exposure times, higher embryo viability and regrowth and greater effectiveness at two pre-treatment temperatures (0°C and 25°C). VIV-cryopreservation may form the basis of a generic, high throughput technology for the *ex situ* conservation of plant genetic resources, aiding food security and protection of species from diverse habitats and at risk of extinction.

## Introduction

The vitrification process plays a key role in cryopreservation for the long term conservation of plant genetic resources [1]. Vitrification is defined as a physical process by which a concentrated aqueous solution solidifies into a stable amorphous glass without the formation of ice crystals when the temperature is decreased [2]. Vitrification of plant specimens can be achieved in many ways, including air drying of embryos [3], and more recently through the use of highly concentrated plant vitrification solutions (PVS) that readily form glasses on cooling and inhibit crystallization [2], [4]. However, exposure to PVS must be controlled to enable sufficient cellular dehydration whilst limiting injury from chemical toxicity and establishment of a simple and high-throughput cryopreservation method using cryoprotectant is highly desirable [5]. Plant vitrification solutions combine cryoprotectants that vary in permeability [e.g. dimethyl sulphoxide (DMSO) and glycerol], such that cellular water is replaced, cell viscosity is increased and the freezing behaviour of the remaining water is altered [6], [7]. PVS2 [2] is probably the most commonly used cryoprotectant (CP) for plant cells, tissues and embryos; for example, the cryopreservation of embryonic axes of citrus [8] and *in vitro* shoot-tips of *Parkia speciosa*, a tropical species with recalcitrant (desiccation sensitive) seeds [9].

The conventional approach to vitrification generally involves a tissue pre-culture step on sucrose-enriched medium, followed by treatment with a loading solution and dehydration, before cooling, with highly concentrated vitrification solution for a period that varies with species, tissue and temperature [10]. For example, 60 min PVS2 treatment was optimal for cryopreserving embryonic axes of *Parkia speciosa* [9]. Moreover, protocorm-like bodies of *Dendrobium* orchid [11] and *Citrus madurensis* embryonic axes [8] required shorter PVS exposure times at 25°C than 0°C, i.e. 20 versus 60 min respectively. Whilst the time window for optimum PVS treatment is wider at lower temperatures, tropical species tend to respond better with warmer temperature treatment, e.g. *Colocasia esculenta* [12].

The physical dimensions (geometry) and permeability characteristics of the tissue under investigation profoundly affect the outcome of the cryoprotection and cryopreservation procedures. Smaller apices (1.5 or 3 mm in diameter) of garlic displayed higher regeneration after cryopreservation than large ones (4.5 mm in diameter) [13]. Similarly, *Nephelium ramboutan-ake* shoot-tips of c. 2 mm tolerated cryopreservation well [14], as did 0.8 mm diameter axillary buds of *Colocasia esculenta* [12]. The cryopreservation of mature zygotic embryos of recalcitrant seeds generally requires a reduction in tissue mass to facilitate cryoprotectant uptake. Usually, this involves the excision of the embryonic axis. In axes of recalcitrant seeds of sweet chestnut (*Castanea sativa*) such surgical intervention results in a burst of superoxide (O_2_^.−^), with further oxidative stress during subsequent desiccation [15]. In this context, the free radical scavenging capability of CPs is additionally crucial for survival of cryopreservation procedures, assuming that the protectants permeate sufficiently. Permeation of chemicals into the intercellular spaces and cells of plant tissues is compounded by many features (e.g. mass, morphology, cellular anatomy and chemical composition). To enable the rapid permeation of the viability stain, triphenyl tetrazolium chloride (TTC), into oily tissues of pine seed, we previously used vacuum infiltration [16]. Similarly, this system has been used to improve efficient gene transformation [17], the delivery of pathogenic bacteria into the intercellular spaces of plants to study pathogen-plant cell interactions [18], [19] and the diffusion of an inhibitor of ethylene action so that pear fruits have prolonged storage [20]. In addition, preliminary studies have shown that vacuum-assisted glycerol cryoprotectant infiltration can preserve the normal histology of rat leg muscle with no ice crystal formation after 3 weeks storage at −80°C [21]. In this study, we developed and compared the efficacy of vacuum infiltration vitrification (VIV) using PVS2 for the cryopreservation of seed embryos of three species with varying morphology, stress physiology and chemistry: *Carica papaya* (Caricaceae); *Passiflora edulis* (Passifloraceae); and *Laurus nobilis* (Lauraceae).

These species have purported differences in seed storage characteristics. *C. papaya* has a high level of desiccation tolerance to about 5% moisture content [22], limited (months) storability at −20°C [23], but tolerance of cryopreservation [24]. *P. edulis* seeds may show reduced viability after drying to 5–6% moisture content, but the majority of dry seeds tolerate cryopreservation [24]. Both species have spatulate embryos in copious endosperm [25]. Finally, *L. nobilis* has seeds with a lowest safe moisture content of c. 24%, below which they are desiccation sensitive and successful moist storage at 0°C is limited to about 4 months [26]. *L. nobilis* embryos are linear and bigger (c. 5 mg dry mass) than both *C. papaya* and *P. edulis*. An unifying feature of these three species is that their seeds contain c. 25% oil. However, fatty acid composition varies greatly. Whilst *L. nobilis* seed has c. 50% saturated fat, mainly laurate (12∶0), *C. papaya* and *P. edulis* seeds are about 80% unsaturated fat with either oleic (18∶1) or linoleic (18∶2) as the main (c. 80%) fatty acid, respectively [27], [28], [29], [30], [31], [32]. Consequently, the thermal behavior of the lipids, and visco-elastic properties of the specimens, should vary between species and this has been correlated with poor dry seed storage performance at c. −20°C in some species, e.g. in an orchid [33] and a few *Cuphea* sp. [34]. Our primary objective in this study is to investigate whether vacuum infiltration vitrification (VIV)-cryopreservation is effective at preserving embryos of species from different provenances (i.e. Mediterranean to tropical), and with disparate morphology, chemistry and physiology. Given that seed desiccation sensitivity in plants correlates strongly with tropical moist forest habitats [35], in which the majority of the world’s plant species grow and which are under continuing threat of deforestation, resolving the *ex situ* conservation options for such species is an urgent imperative.

## Materials and Methods

### Seed Material

Both *C. papaya* and *P. edulis* fruits were purchased from a local supermarket and *L. nobilis* seeds were acquired from a commercial seed supplier. Seeds were extracted from the fruits and surface sterilised with 20% v/v commercial bleach (Domestos) for 10 min before rinsing three times with sterile water.

### Seed and Embryo Desiccation

Seeds of all three species were desiccated to and equilibrated at various moisture contents ranging from c. 40 to 5% (fresh weigh basis) above lithium chloride salt solutions, at various levels of saturation, for up to 2 weeks. Germination was subsequently assessed as radicle emergence (≥2 mm) on 1% (w/v) agar-water. For *L. nobilis*, the embryos (excised and sterilized using the method described below) were also subjected to desiccation to various moisture contents by air drying in a laminar air flow cabinet and germinated *in vitro* in Petri dishes containing MS medium [36]. Seed germination and embryo growth *in vitro* was carried out at 25±2 °C in a temperature-controlled growth chamber with a 16 h light/8 h dark photoperiod (50 µmol m^−2^ s^−1^).

### Embryo Excision and Sterilization

The embryos were excised aseptically and rinsed with 3% (w/v) boric acid for 10 min followed by 1 min in 50% (v/v) ethanol and rinsed again with sterile water. For *C. papaya* the whole embryo was used for cryopreservation, whereas embryonic axes were used for *P. edulis* and *L. nobilis* following removal of the cotyledons by cutting the cotyledonary petiole above the shoot tip. Details of explant size/dimension used are summarised in Table 1.

**Table 1:** Tissue physical and biophysical heterogeneity: dry mass of whole embryo, cotyledon and axis, explants dimension, oil content and tissue lipid melt mid-points for the species studied.

Species	*C. papaya*	*P. edulis*	*L. nobilis*
Whole embryo dry mass (mg)	1.4±0.1^*^	0.9±0.0	4.9±0.5
Cotyledon dry mass (mg)	1.2±0.1	0.7±0.0	3.3±0.5
Axis dry mass (mg)	0.2±0.0	0.1±0.0^*^	1.6±0.3^*^
Seed oil content (%)^§^	28^Ф^	23^ 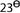 ^	28^ 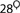 ^
Explants for cryopreservation	Embryo	Embryonic axis	Embryonic axis
Dimension (mm)	3.5×1.0	1.0×0.3	5.6×2.5
Total lipid melt enthalpy (J g^−1^)^#^	26.4±0.6	21.2±1.1	28.1±0.4
Lipid melt 1 mid-point (°C)	−45.2±1.6	−46.2±0.6	−32.4±1.4
Lipid melt 2 mid-point (°C)	−5.4±0.7	−23.8±0.2	−14.2±0.5
Lipid melt 3 mid-point (°C)	10.1±0.5	–	3.1±0.2
Lipid melt 4 mid-point (°C)	–	–	19.0±0.1
Lipid melt 5 mid-point (°C)	–	–	35.1±0.5

For explant dimension measurement, data represent mean ± SE for 4–10 individual embryos/axes; lipid melt peak calculation was based on DSC thermal analysis of embryos/embryonic axes with data shown as mean ± SE for 3 replicates. ^*^Explants used for cryopreservation;^ Ф^ [50]; ^
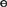
^ [31]; ^
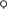
^ [32]; ^§^on fresh weight basis; ^#^on dry weight basis.

### Preculture Treatment

Prior to vitrification treatment, surface disinfected explants were cultured on MS medium supplemented with 0.75 M of sucrose for 1 day at 25±2 °C in a temperature-controlled growth chamber with a 16 h light/8 h dark photoperiod (50 µmol m^−2^ s^−1^). Embryos at this stage served as controls for the vitrification and cryopreservation treatments.

### Vitrification Treatment

The vitrification procedure consisted of dehydrating explants in a loading solution containing 2 M glycerol and 0.4 M sucrose for 20 min at 25 °C, followed by exposure to PVS2 [30% (w/v) glycerol, 15% (w/v) ethylene glycol, 15% (w/v) dimethyl sulfoxide (DMSO) and 0.4 M sucrose] for different durations. For conventional vitrification (CV), explants were treated with PVS2 at 0 and 25°C for 0, 30, 60 and 90 min. For vacuum infiltration vitrification (VIV), the explants were incubated in PVS2 at 0 and 25°C for 0, 1, 2.5 and 5 min. The explants were immersed in 2 mL cryovials containing 1 mL of PVS2 solution in a vacuum chamber (10 L volume) and treated under vacuum (381 mm [15 in] of Hg) for the required period and the vacuum was then released.

### Cryopreservation

Following vitrification treatment, the PVS2 solution was replaced with fresh PVS2 solution at the same temperature, the lid put on to the cryovial and the vial and explants plunged into liquid nitrogen. The explants were stored in liquid nitrogen for at least 24 h. Thereafter, the explants were rapidly warmed by placing the cryovials in a water bath at 38±2 °C for 2 min and then rehydrated for 20 min in liquid MS medium containing 1.2 M sucrose at room temperature.

### Viability and Regrowth Assessments

The control, cryoprotected and cryopreserved explants were cultured on solid MS medium in a temperature-controlled growth chamber at 25±2 °C with a 16 h light/8 h dark photoperiod with light intensity of 50 µmol m^−2^ s^−1^. Explants were transferred to fresh culture medium every day for the first 3 days to reduce any residual toxicity of PVS2. The embryos were considered viable when they turned green, became swollen and started to elongate. The embryos were also assessed for regrowth into seedlings with the first pair of leaves and an extending radicle. Embryo vigour *in vitro* was assessed as the mean time to germinate (MTG) as evidenced by elongation (i.e. viability) in days;


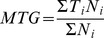
, where 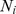 = number of embryos that have elongated at time 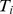 [37].

### Thermal Analysis

Differential scanning calorimetry (DSC) was used to characterise the thermal signature of the seed and embryo lipid and to investigate the presence of free water in the cryoprotected explants in relation to the vitrification potential of the embryos. A DSC 7 (Perkin-Elmer, UK), controlled by a TAC-7 and Pyris 7 software, was calibrated using indium, zinc and pure water as standards for peak temperature and enthalpy. Between 5 to 10 embryos, weighing between 25–35 mg, were placed in pre-weighed aluminium pans, non-hermetically sealed (as recommended for water and lipid phase change studies) with the aid of a Perkin Elmer crimper and weighed to record fresh weight values. Samples were cooled from 25 to −100°C and then rewarmed to 25°C at a cooling/warming rate of ±10°C min^−1^. Dry seeds and embryo/axes (as used in the cryopreservation experiments) with moisture contents ∼4% and without any CP treatment were also subjected to DSC analysis to compare seed and embryo lipid thermal behaviours using the same temperature programme, except for *L. nobilis* which was re-warmed to 50°C. The enthalpies for lipid melting endotherms, and where relevant the enthalpies for ice and PVS2 melting endotherms, were calculated using the Pyris 7 software. When lipid melting events overlapped with the ice or PVS2 melting, the enthalpy for lipid melt (calculated from dry embryos/axes) was subtracted from the total enthalpy to calculate residual melting events. PVS2 solution was also analysed in the DSC during cooling from 25 to −150°C and rewarming to 0°C at a rate of ±10°C min^−1^. All the thermal analyses were replicated three times.

### Statistical Analysis

For each vitrification method, 120 embryos were used in three replicates of 40. The primary response was the percentage of viable (elongating) embryos/axes and the percentage of regrowing seedlings *in vitro*. These percentage values were transformed to logit values, and two-way ANOVA was used to test the level of significance. Means were compared using the Fisher-Pair Wise comparison test. Linear relations between embryo regrowth (%) and mean time for embryo viability (elongation) *in vitro* was assessed for all treatments in GENSTAT. A polynomial fit was used to define the optimal internal PVS2 concentration resulting in maximum post-cryopreservation regrowth using ORIGIN software. To ovoid skewing of the fit, zero values for survival due to ice crystallization and extreme toxicity were excluded.

## Results

### Seed and Axis Desiccation Tolerance

**Figure 1:**
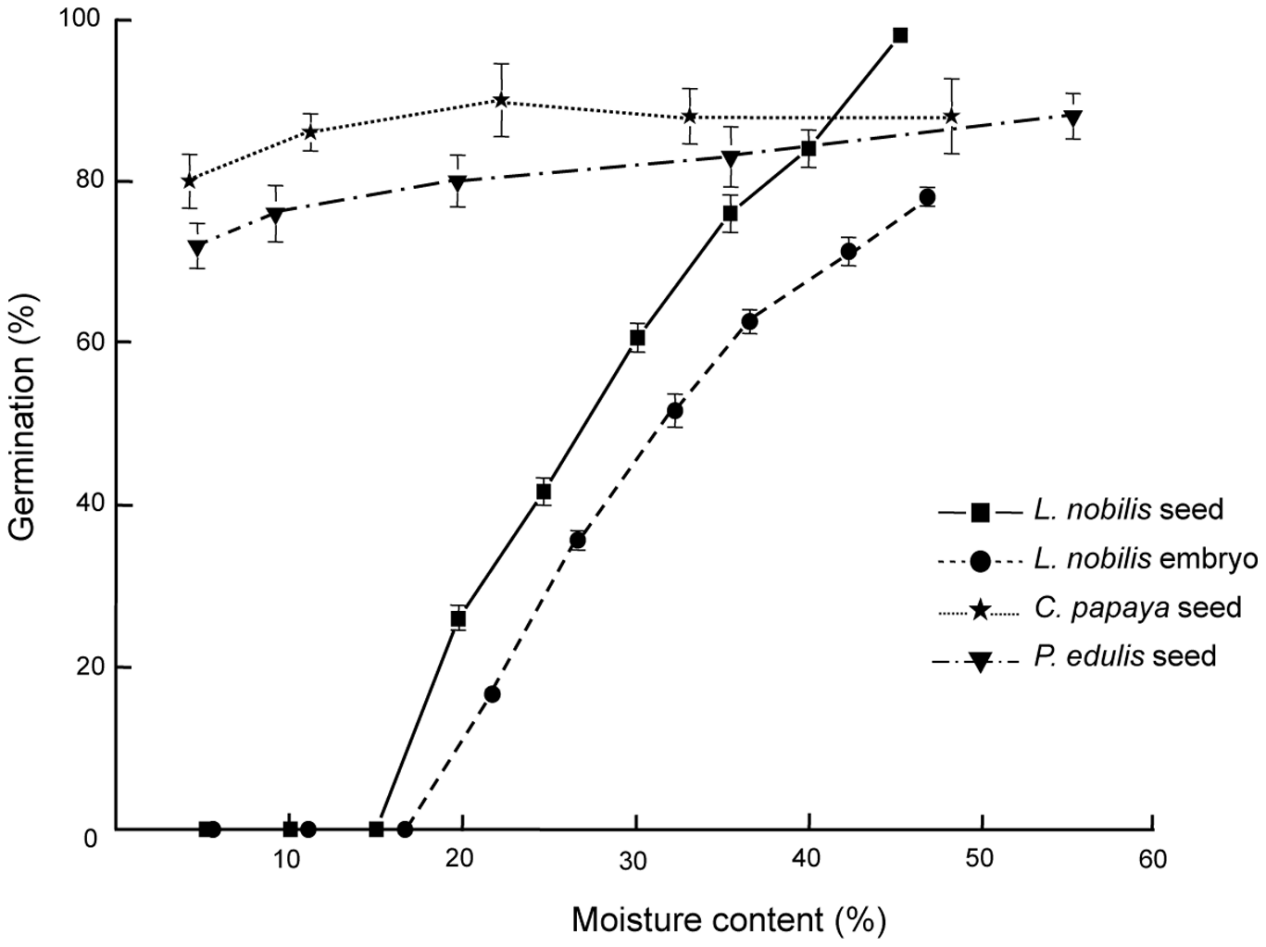
Desiccation sensitivity assessment for seeds and embryos. Legend: Germination percentage of *C. papaya*, *P. edulis* and *L. nobilis* seeds desiccated to various moisture contents and germinated on water agar. Also shown is the *in vitro* germination (regrowth) of *L. nobilis* embryos, following desiccation to various moisture contents, on MS medium at 25±2°C with a 16 h light/dark photoperiod.

Seeds of *C. papaya* and *P. edulis* tolerated desiccation to around 4% moisture content, with germination falling slightly compared to undried material but remaining above 70% (Figure 1). On the contrary, desiccating *L. nobilis* seeds showed a steady decline in germination, with only 40% surviving after drying to 25% moisture content. Seeds lost viability completely by 15% moisture content. Aseptically excised and rapidly desiccated embryos of *L. nobilis* showed a similar trend in decreasing regrowth *in vitro* as the whole seed (Figure 1).

### Effects of Vitrification Method and Temperature

**Figure 2:**
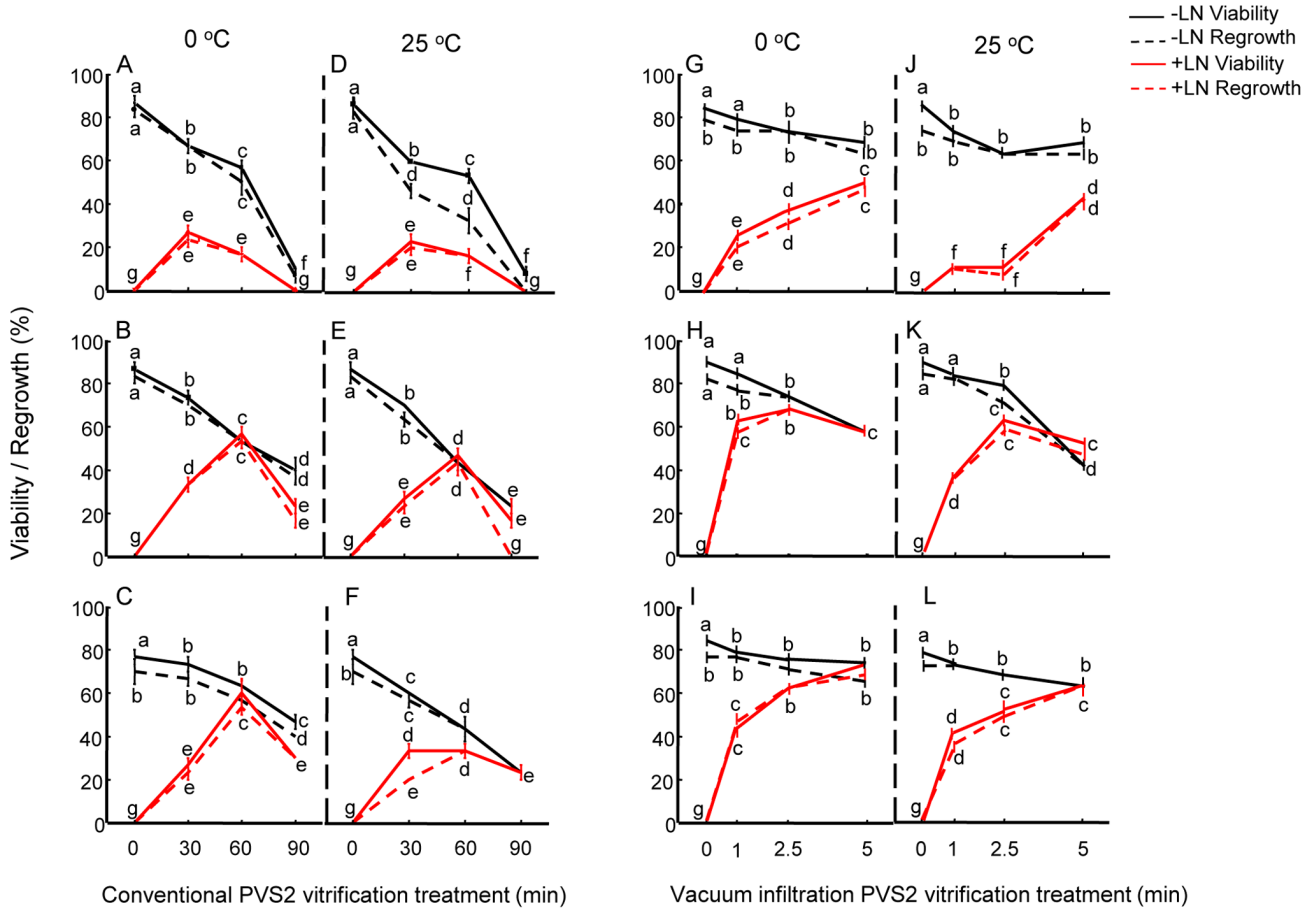
Viability and regrowth of embryos following cryoprotection and cryopreservation. Legend: Viability and regrowth of *C. papaya* (A, D, G, J), *P. edulis* (B, E, H, K) and *L. nobilis* (C, F, I, L) embryos/embryonic axes before (−LN) and after (+LN) cryopreservation using conventional (A–F) and vacuum infiltration (G–L) vitrification at 0°C. Data are means (± SE). Within species, means with the same letters are not significantly different (p<0.05) using Fisher’s pair wise comparison.

With the conventional vitrification (CV) method, embryos of *C. papaya* showed highest post-cryopreservation viability and regrowth (∼25%) with 30 min PVS2 treatment at 0°C (Figure 2A). Increasing the treatment time to 60 and 90 min resulted in lower regrowth of around 18% and no regrowth, respectively. In *P. edulis* axes, highest post-cryopreservation viability and regrowth ∼60% was recorded for 60 min PVS2 treatment (Figure 2B). For *L. nobilis* axes, following cryopreservation, highest viability (60%) and regrowth (52%) was achieved after 60 min PVS2 at 0°C (Figure 2C).

In comparison with CV, the vacuum infiltration vitrification (VIV) method resulted in successful cryopreservation after exposure times to PVS2 about 10-times shorter. Post-cryopreservation viability and regrowth of *C. papaya* embryos increased significantly (p<0.05) to about 55% following 5 min VIV treatment at 0°C (Figure 2G). For the smaller embryonic axes of *P. edulis*, 1 min VIV treatment at 0°C was sufficient for good viability and regrowth (above 55%) following cryopreservation (Figure 2H). Increasing the PVS2 exposure period to 2.5 min increased the regrowth significantly to >63% but then decreased to 50% when the treatment time with PVS2 was extended to 5 min (Figure 2H). In *L. nobilis*, 50% regrowth after cryopreservation was recorded with 1 min VIV at 0°C; regrowth increased significantly (p<0.05) to 60 and 70% following 2.5 and 5 min treatments respectively (Figure 2I).

Because of the subtropical/tropical origin of two of the species, the effect of PVS2 treatment at room temperature was assessed. For *C. papaya*, CV treatment of embryos at 25°C was optimal after 30 min treatment, similar to the findings at 0°C (Figure 2D). However, highest regrowth was still only 20%. VIV for 5 min at 25°C resulted in the highest viability and regrowth (>40%) but regrowth was significantly lower than when applied at 0°C (55%) (Figure 2J). In *P. edulis* axes, 60 min PVS2 CV treatment at 25°C gave the highest viability and regrowth following cryopreservation (Figure 2E); however only 43% regrowth resulted, which was significantly lower (p<0.05) than the 0°C treatment (i.e., 53%). VIV treatment at 25°C for 2.5 min resulted in ∼50% viability and regrowth, but this was significantly (p<0.05) lower than the 60% achieved after 2.5 min exposure at 0°C and cryopreservation (Figure 2H, K). For *L. nobilis* axes, at 25°C, the highest regrowth following cryopreservation was ∼30% after the 60 min CV treatment which was ∼30% lower than that achieved at 0°C (Figure 2C, F). Whilst for this species VIV at 0°C and 25°C for 5 min were optimal, regrowth was significantly lower after the warm temperature treatment (Figure 2I, L).

### Thermal Analysis of the Explants

**Figure 3:**
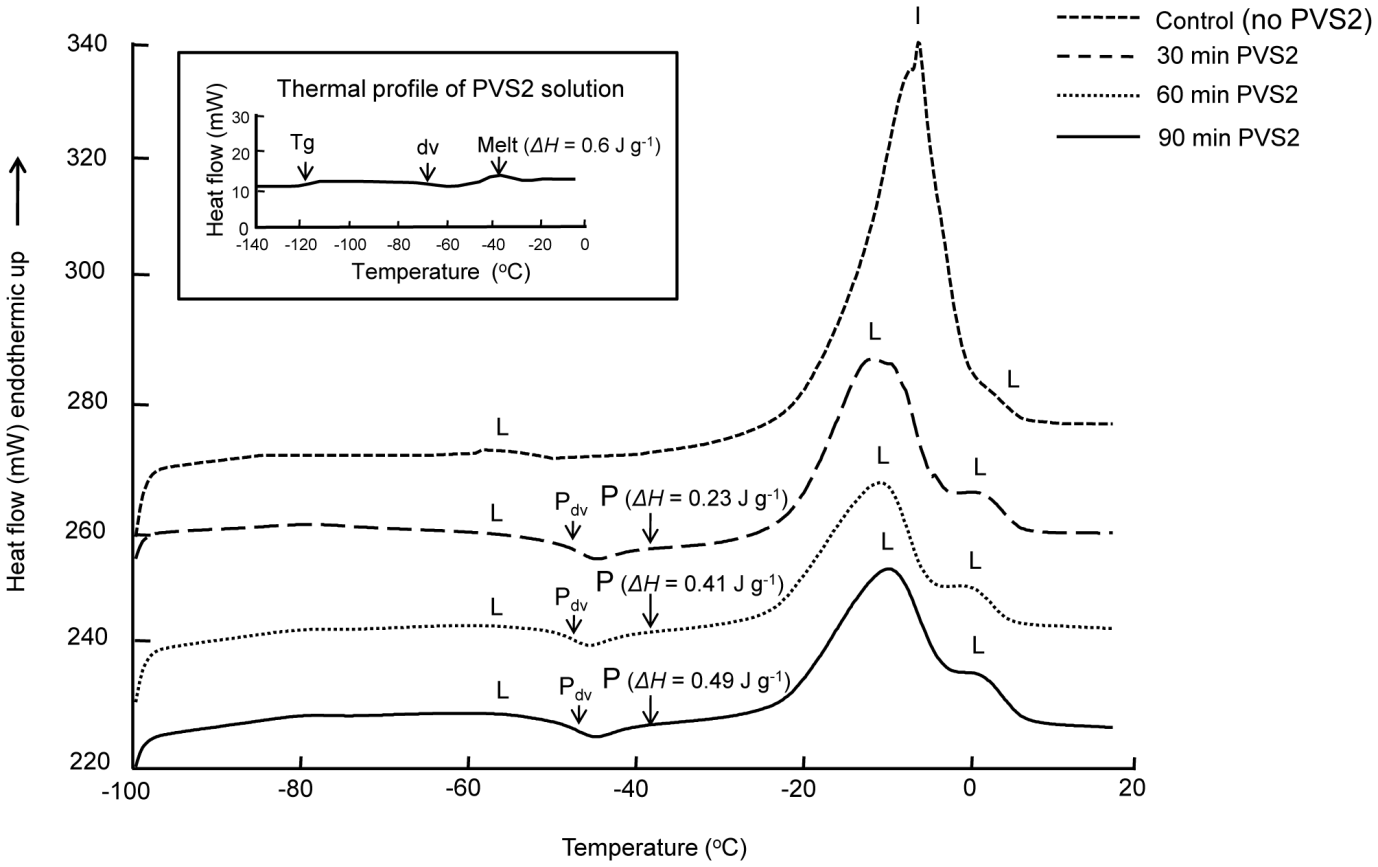
DSC warming thermograms for *C. papaya* embryos following cryoprotection. Legend: Representative DSC warming thermograms for *C. papaya* embryos untreated and treated with PVS2 under CV for 30, 60 and 90 min at 0°C showing ice melt (I), lipid melt (L), PVS2 melt (P) with melt enthalpies in brackets and PVS2 de-vitrification (P_dv_). Samples were warmed from −100 to 25°C at 10°C min^−1^. Inset: Warming thermal profile of PVS2 solution with melt enthalpy in bracket (warming program: from −150 to 0°C at 10°C min^−1^).

Following PVS2 treatments, endotherms during warming were interpreted for different CV and VIV treatments, so that cryopreservation procedures could be optimised in relation to the presence/absence of freezable water and the permeation of PVS2. For all the species, there was a large ice melting peak observed in embryos not treated with PVS2 [e.g. for *C. papaya* (Figure 3)]. This peak was lost for *C. papaya* embryos, after 30, 60 and 90 min CV treatments at both temperatures, indicating freezable water had been removed from the embryos (Figure 3). However, whilst 60 and 90 min CV treatments avoided ice formation, the toxicity effect of the PVS2 was already evident in terms of reduced viability and regrowth (Figure 2A, D). For VIV at both temperatures, a small ice melting peak was observed for the 1 and 2.5 min treatments showing this exposure time was unlikely to achieve a vitrified state without any ice formation; consequently, regrowth was relatively low (Figure 2G, J). Extending the VIV treatment to 5 min resulted in no ice melting peak and the highest viability and regrowth following cryopreservation. For *P. edulis* axes, no ice melting was observed after all three exposure times for both CV and VIV at both temperatures. In the case of *L. nobilis* axes, 30 min CV treatment was insufficient to avoid the presence of an ice melt, but this was lost when 60 and 90 min exposure times were used at both temperatures. For VIV treatment, all three exposure times showed thermal profiles with no ice melting peak.

**Figure 4:**
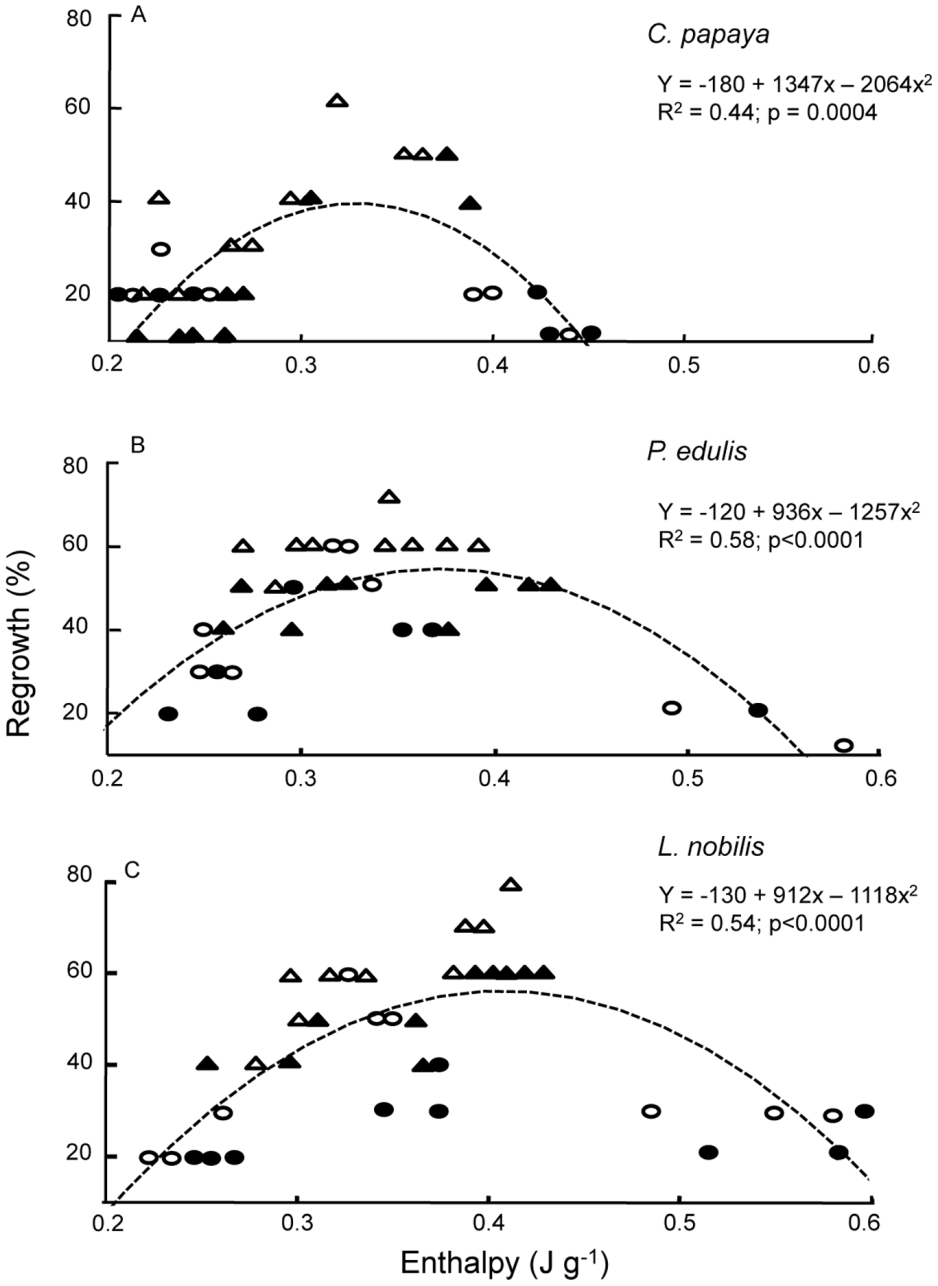
Embryos internal concentration of PVS2 and their corresponding regrowth. Legend: Regrowth following cryopreservation compared to internal concentration of PVS2 (defined by melt enthalpy using a DSC) of *C. papaya* (A), *P. edulis* (B) and *L. nobilis* (C) embryos/embryonic axes with a polynomial fit. (**○** = CV at 0°C; • = CV at 25°C; Δ = VIV at 0°C; ▴ = VIV at 25°C).

The thermal profile of PVS2 solution during warming is presented as an inset in Figure 3. A glass transition (Tg) was observed at −115°C, followed by a devitrification (recrystallization) at around −75°C. The solution started to melt around −50°C with a midpoint of −37°C. The enthalpy of the PVS2 solution melt was 0.6 J g^−1^. The enthalpy of PVS2 melt in CV-treated *C. papaya* embryos increased from 0.23 to 0.41 and to 0.49 J g^−1^ as PVS2 treatment time was extended from 30 to 60 and to 90 min (Figure 3 inset). A polynomial fit for PVS2 melt enthalpy in explants and their regrowth revealed that the estimated optimum internal PVS2 was approximately 55 to 67% (0.33 to 0.4 J g^−1^ melting) for all three species (Figure 4A–C).

**Figure 5:**
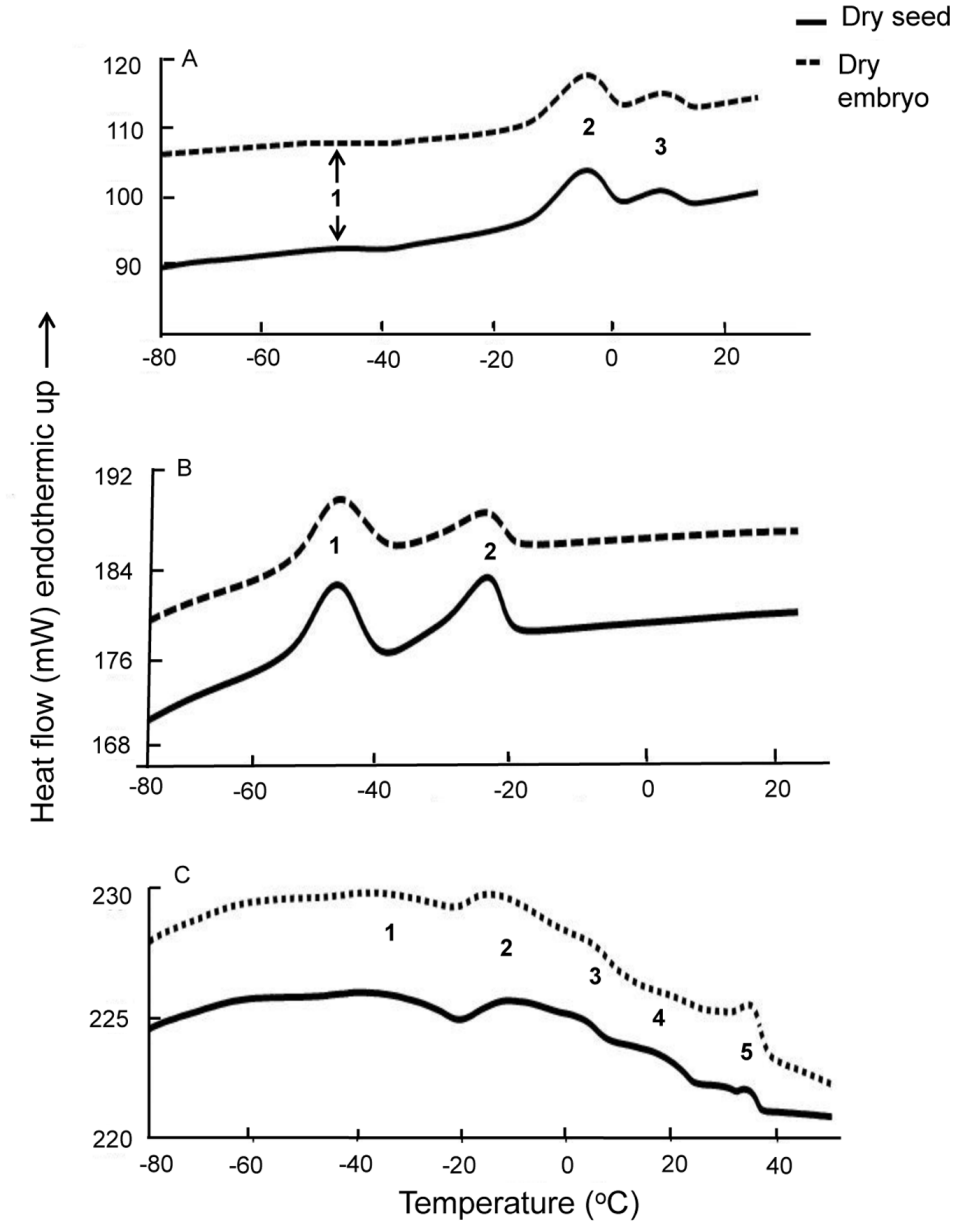
DSC thermograms for dry seeds and embryos. Legend: DSC warming thermograms for *C. papaya* (A), *P. edulis* (B) and *L. nobilis* (C) dry seeds and embryos/embryonic axes showing lipid melt endotherms. Samples were cooled from 25°C to −100°C and rewarmed to 25°C (50°C for *L. nobilis*) at ±10°C min^−1^. Numbers on each peak indicating different lipid melt peaks corresponding to peaks in Table 1.

As the visco-elastic properties of explants may affect cryoprotectant permeation and cryopreservation success, the thermal fingerprints for dry seeds and embryos were also characterised. Three prominent melting peaks were observed for *C. papaya* dry seeds and embryos at −45, −5 and 10°C indicating the embryos have relatively high lipid contents (Table 1; Figure 5A). Based on the published literature, the small melting peak at −45°C is consistent with linoleic acid (c. 3% of lipid in this species), and the combined peaks 2 and 3 at −10 to 10°C likely correspond to the presence of oleic acid (c. 65% of seed lipid). For the dry seed and embryonic axis of *P. edulis*, two main melts were observed at −45°C and −23°C, consistent with the presence of linoleic (74% of lipid) and oleic (17% of lipid) acids (Figure 5B). Several lipid melt peaks were evident for *L. nobilis* dry seed/axis (Figure 5C). Melting around 35°C was assigned to lauric acid (c. 38% of lipid). Other peaks, at −38°C and −15 to 15°C, correspond to the presence of linoleic (c. 40%) and oleic acids respectively. The results indicate potentially large differences in visco-elastic properties of the explants during the cryopreservation procedures at 0°C and 25°C.

### Embryo Vigour

**Figure 6:**
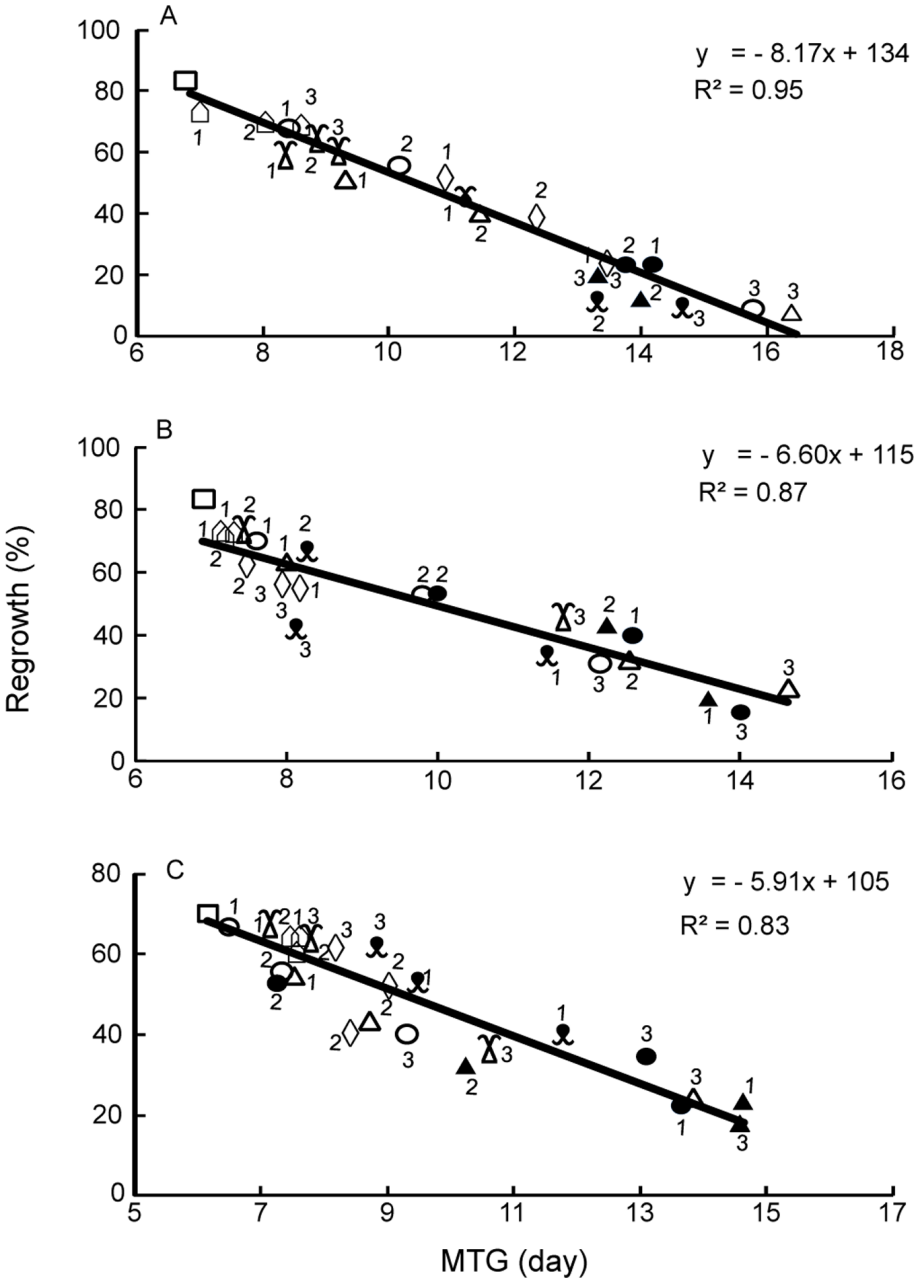
Seedling vigour and stress. Legend: Mean time to germinate (MTG) based on embryo viability (elongation) compared to regrowth for *C. papaya* (A), *P. edulis* (B) and *L. nobilis* (C) showing relationship between seedling vigour and stress. (□ = control; ○ = CV at 0°C -LN; • = CV at 0°C +LN; Δ = CV at 25°C –LN; ▴ = CV at 25°C +LN; 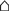 = VIV at 0°C -LN; ◊ = VIV at 0°C +LN; 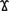 = VIV at 25°C –LN; 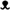 = VIV at 25°C +LN). Numbers by each symbol represent: 1 = 30 min; 2 = 60 min; 3 = 90 min for CV; and 1 = 1 min; 2 = 2.5 min; 3 = 5 min for VIV respectively.

In addition to recording embryo viability and regrowth percentages following cryopreservation, embryo vigour was assessed by measuring embryos/axes elongation (viability) *in vitro* over time. Linear relations were found for all three species between the percentage of embryo regrowth and the mean times for elongation (MTG) in days (Figure 6A–C). Accumulation of stress following PVS2 and cooling/warming treatments were very evident for *C. papaya* embryos; for both vitrification treatments, MTG increased from 7 days for the control to 8 days following cryoprotection and 14 days following cryopreservation for both treatment temperatures (Figure 6A). However, for *P. edulis* and *L. nobilis* axes, the MTG increased from c. 6 days (control) to c. 15 days following CV. In contrast, MTG remained at 8 days after VIV cryoprotection and cryopreservation, irrespective of treatment temperature, indicating the imposition of minimal stress by VIV (Figure 6B, C).

## Discussion

The successful cryopreservation of seed embryos is determined by a complex interaction of factors that contribute to sample heterogeneity. These include structure (morphology and ultrastructure), cell physiology and stress (desiccation, cold) tolerance, and chemical composition and associated thermal properties, all of which impact on the optimisation of cryoprotection (permeation time and toxicity) and cryopreservation procedures (cooling/warming) [2], [9], [10], [38], [39].

Desiccation tolerance, as measured by seed germination and embryo recovery *in vitro*, varied for the three species studied. *C. papaya* tended to an orthodox response to drying, with 80% germination after desiccation to 5% moisture content and is in agreement with earlier findings [22], [40]. However, the seeds store poorly under conventional seed bank conditions (−20°C) and therefore cryopreservation is recommended for long-term seed/embryo storage [23], [24], [41], [42]. A similar level of drying tolerance was observed in *P. edulis* seeds, related species of which are also sensitive to dry, cold (−20°C) storage [43], [44]. In comparison, *L. noblis* seeds are highly sensitive to drying, with reduction of germination to 50% (LD_50_) at ∼30% moisture content (Figure 1). Whilst desiccation sensitivity in *L. nobilis* seeds has been noted previously [45], [46], [47], [48], and loss of viability within one year’s moist storage [26], our study is the first detailed characterisation of seed and embryo desiccation sensitivity for this species.

Variation in biophysical properties of the embryos was also evident for the three species, specifically in their lipid thermal fingerprints, as determined by DSC, the peak temperatures for which spanned 83°C [−45°C (onset) to 38°C (endpoint)]. In accordance with Hor *et al.* [39] and Hamilton *et al.* [49] who assigned particular thermal properties in oilseeds (and their embryos) to individual lipid/fatty acids in *Citrus*, melting peaks at −45°C in all three species is assigned here to linoleic acid: 3% of seed oils in *C. papaya*, 74% in *P. edulis* and 40% in *L. nobilis* [29], [50], [51]. For the three species, melting peaks between about −20 and 15°C occur as single or complex peaks (i.e. with shoulders) and have been assigned previously to oleic acid, e.g. in *C. papaya* [50] and *L. nobilis* [51]. Oleic acid is generally present in plants/seeds as a mono-unsaturated omega-9 fatty acid (18∶1 *cis* 9). However, this fatty acid is known to be susceptible to oxidation and to be less thermodynamically stable than the *trans* forms [52], perhaps explaining why we observed more than one peak in *C. papaya* and in *L. nobilis*. Finally, one of the main fatty acids in *L. nobilis* is lauric acid (38% of seed lipid; [53]) which we observed to melt at c. 35°C. This thermal response is similar to that seen in a number of *Cuphea* species that have high laurate and capric acid contents [54]. Consequently, seeds of these three species stored at any temperature from c. 38 to −45°C will be in different physical states, which may impact on their storage (in)stability [33], [34], [54] and also ensures variation in embryo visco-elastic properties during cryoprotection at the two pre-treatment temperatures used in this study.

The prepared explants (embryos and embryonic axes) of the three species differ in physical dimension, with *L. nobilis* axes being about 10 times heavier and larger than those of *P. edulis*. Consequently, a single treatment with cryoprotectant (e.g. time×temperature) is unlikely to result in an equal level of permeation and thus protection due to variable surface-area-to-volume ratios and diffusion path lengths. Often, PVS2 treatment of explants ≤0.5 mm diameter risks toxicity and explants ≥3 mm require much longer times for effective dehydration [55]. Our observation showed that for *L. nobilis* and *P. edulis* with large and small axes respectively, cryopreservation was maximal after 60 min CV PVS2 treatment at both 0 and 25°C. In comparison, whole *C. papaya* embryos, which are slightly larger than *P. edulis* axes, required a shorter PVS2 exposure time (i.e. 30 min). Overall the results show that tissue mass and volume are not the only determining factors for optimising PVS2 treatment duration. Possibly, not removing the spatulate cotyledons from *C. papaya* embryos may have enabled cryoprotectant permeation into the axis.

Maximal cryo-survival is presumed to coincide with an optimum internal concentration of cryoprotectant agent (CPA), which is generally not known for the sample under investigation, and the avoidance of ice formation in the tissues. According to our observation (Figure 3, inset) and Sakai *et al.* [56], PVS2 solution has a melting peak at ∼−36°C. For *C. papaya,* with little linoleic acid (∼3%) melting at −45°C, PVS2 melting during rewarming was evident as a small but distinct peak centred at −37°C (Figure 3). This peak was absent in control embryos (i.e. without PVS2 treatment). The enthalpy of the PVS2 melt in the embryos increased with treatment time (Figure 3). At the optimum PVS2 treatment time of 30 min for CV and 5 min for VIV, the enthalpies of the PVS2 melt in *C. papaya* embryos were approximated to 48% and 58% respectively of the maximal concentration, based on the total enthalpy of PVS2 solution (Figure 4A). Further increase in internal concentration of PVS2 to 75% of the signal intensity of full strength PVS2, as a result of extending the treatment to 90 min, resulted in no post-cryopreservation viability, presumably as a consequence of cytotoxic effects. Interestingly, *C. papaya* embryos showed some post-cryopreservation survival following 1 and 2.5 min VIV treatments even though the DSC thermograms indicated small ice melting peaks for these cryoprotected embryos. This was also the case for *L. nobilis* axes treated with 30 min CV. In these cases, water crystallisation may have occurred in non-critical tissues, e.g. extracellularly, or ice formation limited to those embryos that failed to tolerate cryopreservation, i.e. a population effect. A similar phenomenon of ice formation was observed in surviving shoot-tips of *Parkia speciosa* following encapsulation, 60 min PVS2 exposure and cryopreservation [9] and in *Acer saccharinum* embryonic axes [57].

When the average total lipid melting enthalpies of *P. edulis* and *L. nobilis* axes were accounted for, it was possible to assign the residual signal to PVS2 melting. For the embryos/axes of the three species, the average internal optimum PVS2 concentration for cryopreservation success is estimated to correspond to 55–67% of maximal concentration (i.e. on a J g^−1^ basis c.f. 0.6 J g^−1^ for PVS2 solution). Personalisation of vitrification solution concentrations to balance osmotic and toxicity effects has been recommended by Kim and Lee [58], on the basis of improved cryopreservation of plant cells using diluted PVS. For the three species tested here, 60% concentration of PVS2 is equivalent to a water potential around −31 MPa. This approximates to the water potentials at which recalcitrant seeds have lost all free (and thus freezable) water, i.e. −30 MPa [59], and close to the optimum for the cryopreservation of desiccation sensitive seeds of four citrus species, i.e. −38 MPa [39]. Consequently, it appears that for the successful cryopreservation of diverse embryos of various plant taxa a precise internal concentration of cryoprotectant, of c. 60% PVS2, may be required.

The uptake of CPAs in animal and human tissues is enhanced with increasing temperature; for example in rat kidney cortex and rat liver [60], medaka fish embryos [61] and human oocytes [62]. For DMSO, enhanced uptake at higher temperatures may be due to the progressive destabilization of the phospholipid bilayers [63], which increases the risk of toxicity [64], greater oxidation of sulfhydryl groups [65] and, in the presence of glycerol, the formation of formaldehyde by non-enzymatic reactions [65]. Nonetheless, in many tropical and sub-tropical plant species, regrowth is better after cryopreservation when PVS2 is applied at ambient temperature [55], as lower temperatures may cause chilling stress [8], [66]. In contrast, most woody, herbaceous temperate and some sub-tropical plant tissues tolerate PVS2 at 0°C [2], but optimum exposure times are longer compared to room temperature application, e.g. 20 min at 25°C and 60 min at 0°C for *Citrus madurensis* embryonic axes [8]. Whilst the optimum PVS2 treatment of 60 min resulted in similar viability after cool or warm application in *C. papaya*, *P. edulis* and *L. nobilis* responded better to cryoprotection under cool conditions, both in terms of viability and vigour. Thus, the embryos of the three species investigated are relatively chilling tolerant over the relatively short, optimum time intervals used in the CV study (≤1 h).

One question we sought to answer was whether a more uniform and faster permeation of PVS2 was possible, such that the obvious inter-species and tissue-specific variables of time×temperature×concentration effects could be reduced and cryo-survival increased. Vacuum degassing at 96 kPa has been used on seeds to enable faster permeation of the viability stain triphenyl tetrazolium chloride into whole pine seeds [16]. A weaker, ‘soft’ vacuum ( = 50 kPa) has also been used in gene transformation studies in one-week old tobacco seedlings [67]. We opted to use ‘soft’ vacuum for isolated embryos/axes, resulting in ∼10-fold reduction in optimum PVS2 exposure times to 2.5 or 5 min for the VIV approach, compared with 30–60 min for the CV method (Figure 2). Moreover, the overall regrowth level was higher and recovery growth was faster (Figure 6) following VIV-cryopreservation. Generally, increasing pressure enhances permeability of cells and tissues, e.g. K+ permeability into human red blood cells [68] and DMSO uptake into 8-cell medaka fish embryos [61]. However, plant tissues contain interconnected networks of intra-cellular (symplast) and inter-cellular (apoplast) spaces, the latter being air-filled at most times in live tissue [69], [70]. In detached roots of maize, the threshold pressure for infiltration of these spaces is about 40 kPa [71]. Over short exposure times we contend that the intercellular spaces could generate short-range thermodynamic disequilibration between the inside of the tissues and in the external solution by maintaining a (short-term) pressure difference (solute equilibrium pressure differential), as suggested on theoretical grounds by Elmoazzen *et al.* [72]. A vacuum environment would eliminate any pressure differential in the tissue, by removing air from the inter-cellular spaces, thus enabling tissues to expand rapidly with the inflow of CPA from the surrounding solution. For the embryos of the three species investigated, VIV permits successful cryopreservation after PVS2 treatments of a few minutes, and irrespective of the differing embryo morphologies (embryo vs axis, smaller vs larger). Indeed for the smallest embryo tissue used (axes of *P. edulis*), DSC showed that free water was removed from the tissues by 1 min VIV. Whilst a preliminary report has shown the preservation of normal histology of rat let muscle tissue after vacuum-assisted cryoprotectant infiltration [21], we believe our studies on three species provide the first report of fully functional tissue survival and regrowth after VIV-cryopreservation. There is an additional significance to this finding, as the VIV method may have overcome the further challenge of cryoprotectant permeation into oily, and thus relatively hydrophobic, embryos.

The restoration of tissue function is critical to successful cryo-conservation [73] with each step of the cryopreservation protocol having the potential to introduce stress: oxidative burst at embryo excision [15], shoot-tip damage by plasmolysing cryoprotection [74], cooling/warming and recovery procedures [9]. Ultimately, stress may be so great that only callus growth ensues after cryopreservation, e.g. embryos of some recalcitrant seeds [75]. Embryo regrowth *in vitro* after cryopreservation was observed in embryos/axes of all three species investigated (Figure 2) and speed of initiation of elongation (vigour) shown to be negatively related to regrowth level (Figure 6). The value of such an analysis of vigour is that the impact to viability of any single step in the cryopreservation protocol is observable as a continuum of stress effects across the population of samples.

In conclusion, VIV-cryopreservation has advantage over CV by shortening cryoprotectant treatment times to a few minutes, reducing the temperature dependency for cryoprotectant application and enabling higher post-cryopreservation regrowth (level and rate). Another advantage of this technology is the reduced risk of cross-contamination of specimens during liquid nitrogen storage compared to ultra-rapid cooling methods that bring the tissues into direct contact with the cryogen (e.g. the propelling of axes into cryogen slurry, droplet vitrification) as the specimens are enclosed in cryovials. As VIV-cryopreservation also overcomes the challenges of specimen heterogeneity (mass, volume, oil composition and thermal properties), the method shows considerable promise as the basis of a generic method for the preservation of various tissues of plant species.
